# Cu Precipitation Behaviors and Microscopic Mechanical Characteristics of a Novel Ultra-Low Carbon Steel

**DOI:** 10.3390/ma13163571

**Published:** 2020-08-13

**Authors:** Mingxue Sun, Yang Xu, Tiewei Xu

**Affiliations:** School of Mechanical and Automotive Engineering, Qingdao University of Technology, Qingdao 266520, China; xy45269026@gmail.com (Y.X.); twxu@qut.edu.cn (T.X.)

**Keywords:** cooling rate, Cu precipitation, Vickers hardness, nano-hardness

## Abstract

We studied the effect of Cu addition on the hardness of ultra-low carbon steels heat treated with different cooling rates using thermal simulation techniques. The microstructural evolution, Cu precipitation behaviors, variations of Vickers hardness and nano-hardness are comparatively studied for Cu-free and Cu-bearing steels. The microstructure transforms from ferritic structure to ferritic + bainitic structure as a function of cooling rate for the two steels. Interphase precipitation occurs in association with the formation of ferritic structure at slower cooling rates of 0.05 and 0.2 °C/s. Coarsening of Cu precipitates occurs at 0.05 °C/s, leading to lower precipitation strengthening. As the cooling rate increases to 0.2 °C/s, the interphase and dispersive precipitation strengthening effects are increased by 63.9 and 50.0 MPa, respectively. Cu precipitation is partially constrained at cooling rate of 5 °C/s, resulting in poor nano-hardness and Young’s Modulus. In comparison with Cu-free steel, the peak Vickers hardness, nano-hardness and Young’s Modulus are increased by 56 HV, 0.61 GPa and 55.5 GPa at a cooling rate of 0.2 °C/s, respectively. These values are apparently higher than those of Cu-free steel, indicating that Cu addition in steels can effectively strengthen the matrix.

## 1. Introduction

Ultra-low carbon Cu bearing high strength steels have drawn extensive attention for many years. In such steels, the addition of Cu can give a significant improvement on the strength by means of precipitation strengthening. The microstructural evolution and mechanical properties have been widely studied by previous researchers [1,2,3,4,5]. Nano-sized Cu precipitates were mainly obtained during tempering process, thus Cu precipitation behavior and its effect on mechanical properties have received a large number of concerns. Mujahid [6] investigated the influence of aging heat treatment on microstructure and mechanical properties in HSLA-100 steels at aging temperatures between 450 and 730 °C. The peak in hardness, yield strength and tensile strength corresponded to the formation of a large number of coherent Cu precipitates at aging temperature of 450 °C. Thereafter, due to the coarsening of Cu precipitates and recovery of the matrix, the impact toughness improved continuously at expense of strength. Dhua [7] and Hwang [8] comparatively studied directing quenching and tempering (DQ and T) as well as reheat quenching and tempering (RQ and T) techniques for Cu-bearing high strength steels. The finer lath martensitic structure, smaller Cu and Nb (C, N) precipitates obtained in DQ and T process contributed to the high strength and good impact toughness. Recently, many studies offered a new strategy to improve the thermal stability of Cu precipitates. The precipitate consisted of a Cu-enriched body-centered cubic (bcc) core and a B2-NiAl/Ni (Al, Mn) shell, leading to the high strength of the steel without sacrificing the ductility [9,10,11,12,13].

With the development of the industry, thermo-mechanical controlled process (TMCP) has been extensively applied to heavy gauge plates with excellent mechanical properties in the recent years. For hot rolled heavy gauge plates, different cooling rates appeared in the surface and center of the steel plates, resulting in non-uniformity of the mechanical properties. This property non-uniformity can be improved by Cu precipitation strengthening formed during slow cooling in the center of the steel plate, which can provide a new idea for developing heavy gauge plates. Thus, study of Cu precipitation behaviors during continuous cooling process can provide an important theoretical support to produce these steels. However, Cu precipitation formed during the cooling process has not been explored much [14,15,16,17]. During cooling stage, Cu precipitation occurs by dispersive precipitation and interphase precipitation mechanisms. It has been suggested that interphase precipitation occurred only at a certain range of cooling rates and above some critical temperatures. Liu [14] reported that the interphase precipitation took place only in association with the formation of polygonal ferrite at a slow cooling rate of 0.1 °C/s, while Cu precipitation during aging process can be achieved during and after the formation of acicular ferrite at high cooling rate of 10 °C/s. Thompson [15] demonstrated that Cu precipitation mainly occurred via interphase precipitation during continuous cooling in A710-type steels. Precipitate-free could be observed at high temperatures, whereas interface precipitation formed at the interfaces between polygonal ferrite/Widmanstätten ferrite and austenite at lower temperatures.

Cu precipitation strengthening effect can be influenced by precipitation type, number density, particle size and distribution. Most of the previous studies investigated the precipitation strengthening by use of Vickers hardness and tensile testing, in which the strengthening effects by grain boundaries had been inevitably included [18,19,20]. It has been proved that nanoindentation is an effective method to preciously measure mechanical properties, such as nano-hardness, Young’s Modulus, residual stresses and residual stresses [21,22]. In comparison with traditional mechanical property testing methods, nano-indentation can provide continuous nanohardness-indentation depth curves, which could directly reflect the effect of Cu precipitation on mechanical properties [23,24,25]. Therefore, Cu precipitation strengthening effect can be fully explored using nano-indentation technology.

In the present study, the influence of Cu addition on microstructural evolution, Vickers hardness and nano-hardness was comparatively studied using optical microscopy, transmission electron microscopy (TEM), Vickers hardness tester and nano-indenter. The morphological evolution of Cu precipitates and its influence on hardness were analyzed. It is expected to provide some technical supports for developing high strength Cu-bearing steels.

## 2. Experimental Procedure

**Figure 1:**
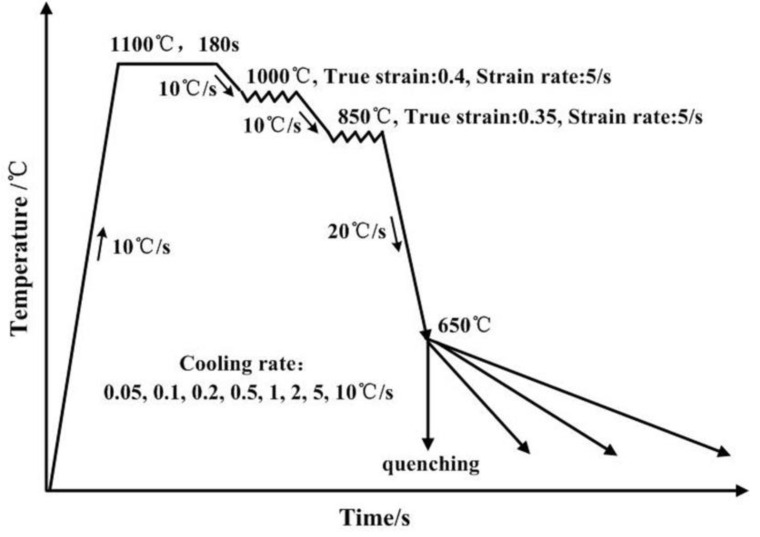
The schematic illustration of the thermal simulation experiments.

In this study, two steels were designed as given in Table 1. The bulk composition of the steels was determined by chemical composition analysis at the Analysis and Test Center of Northeastern University (Shenyang, China) following ASTM A751 standard [26]. In order to eliminate the interference of carbide precipitation, the carbon concentration was ultra-low in the two steels. Meanwhile, the ultra-low carbon concentration can provide excellent welding property and low temperature toughness. Based on the composition characteristics, the two steels were referred as Cu-free steel and Cu-bearing steel, respectively. The tested steels were cast into 50 kg ingots by vacuum melting, and forged into billets with a size of 80 × 80 × 120 mm^3^. The billets were hot rolled into 12 mm in thickness by use of a 450 mm mill. In order to homogenize the alloying elements, the plates were solution-treated at 1100 °C for 24 h and quenched to room temperature. Cylindrical samples for thermal simulation experiments were cut from the slabs along the rolling direction, and then machined into dimensions of Φ 8 mm× 15 mm. The thermal simulation experiment was conducted in a MMS-300 model dynamic thermo-mechanical simulator (State key laboratory of rolling and automation, Shenyang, China). A schematic illustration is shown in Figure 1. Specimens were homogenization-treated at 1100 °C for 180 s with a heating rate of 10 °C/s, followed by a two-stage deformation. The true strain was 0.4 in the first stage at 1000 °C and 0.35 in the second stage at 850 °C, with a same strain rate of 5 s^−1^. Subsequently, the specimens were cooled to 650 °C at a cooling rate of 20 °C/s, followed by cooling to room temperature. The cooling rates were varied between 0.05 and 10 °C/s for studying the effect of cooling rate on Cu precipitation behaviors. Specially, a sample of Cu-bearing steel was directly quenched from 650 °C to room temperature to fully investigate the Cu precipitation behaviors.

Specimens of thermal simulation experiments were cut along the radial direction. The mechanically polished specimens were etched in 2 vol% nital, and then observed on a LEICA-DMIRM Q550 (LEICA Microsystems, Wetzlar, Germany) model optical microscope. The micro-hardness examination of ferrite was conducted in a FM-700 Vickers hardness tester (Future-Tech, Kawasaki, Japan). The loading force was 20 g, loading time was 10 s, and the average value was calculated by detecting 20 positions. For nano-indentation experiment of ferrite, the samples were electrochemically polished by a mixture of 13% perchloric acid, 6% deionized water and 81% ethanol, with a potential of 28 V and time of 25 s. The experiments were carried out in a triboindenter in-situ nano-mechanical testing system (Hysitron, Minneapolis, MN, USA) with a three-sided pyramidal Berkovichtip. A 5-5-5 loading mode (loading time for 5 s, holding time for 5 s, unloading time for 5 s) was used, and the loading force was 2000 μN. The twin-jet electropolished specimens were examined in a Field Electron and Ion (FEI) Tecnai G^2^ F20 field emission type TEM (FEI, Hillsboro, OR, USA). Thin slices of ~50 μm were prepared using sandpapers, and then electrolytically thinned in a 8 vol% HCLO_4_ + 92 vol% C_2_H_5_OH solution.

## 3. Results

**Figure 2:**
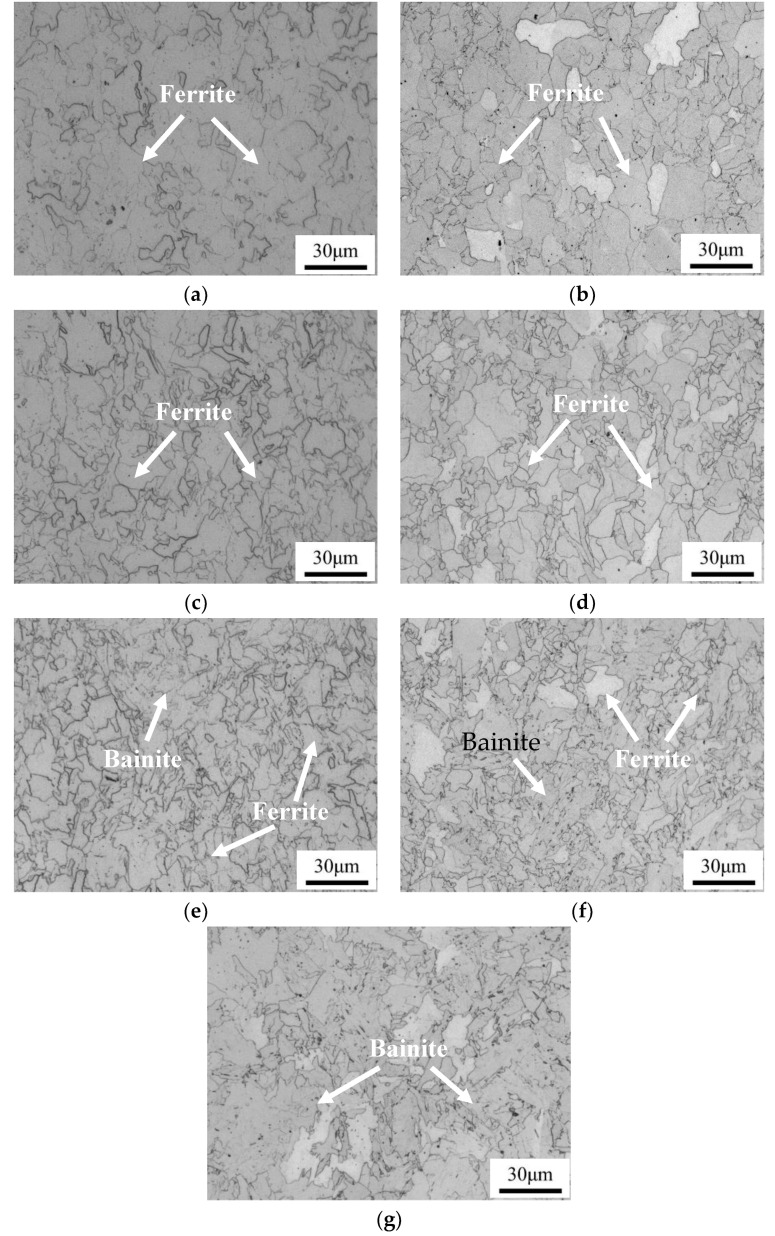
Optical microstructure of Cu-free and Cu-bearing steels at different cooling rates. (**a**,**c**,**e**) 0.05, 0.2 and 5 °C/s for Cu-free steel, respectively, (**b**,**d**,**f**) 0.05, 0.2 and 5 °C/s for Cu-bearing steel, respectively, (**g**) as-quenched condition for Cu-bearing steel.

Figure 2 shows the optical microstructure of the tested steels at different cooling rates. It can be seen that the increase of cooling rate leads to a significant change in the microstructure for the two steels, i.e., from ferritic structure to ferritic + bainitic structure. This result stems from the phase transformation shifting toward the lower temperature region as the cooling rate increases. Additionally, due to the very fast cooling rate in quenching process, no ferritic structure can be found in the as-quenched specimen of the Cu-bearing steel. Using a linear interception method, the average sizes of the ferrite grains in Cu-bearing steel are measured to be 7.8 ± 1.4 μm, 6.8 ± 1.6 μm and 6.0 ± 1.4 μm at cooling rate of 0.05, 0.2 and 5 °C/s, respectively. The corresponding values are measured to be 8.5 ± 1.6 μm, 7.2 ± 1.2 μm and 6.4 ± 0.7 μm for Cu-free steel. Five images were used for each process by use of Image-Pro Plus software. It can be seen that the microstructure is finer in the Cu-bearing steel, owing to the inhibition of nucleation and growth of ferrite.

**Figure 3:**
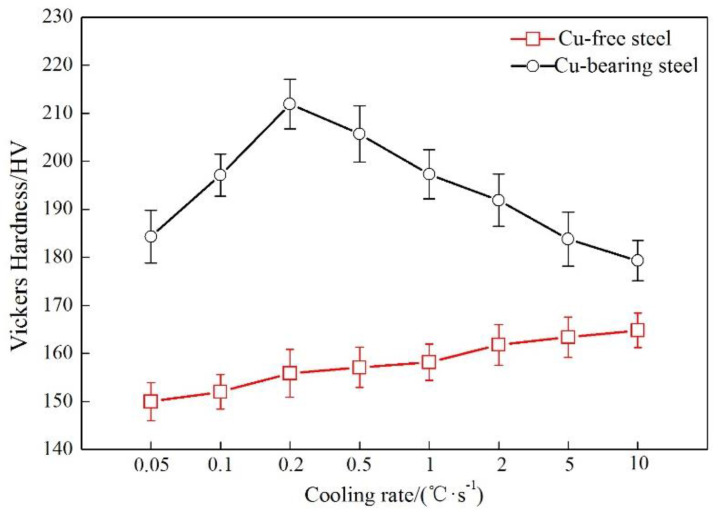
Variation of Vickers hardness of ferrite as a function of cooling rate for the two steels.

As Figure 3 shows, there are big differences in the variation of Vickers hardness of ferrite for the two steels. The Vickers hardness rises slowly with the increase of cooling rate for Cu-free steel, showing the hardness value from 150 HV at 0.05 °C/s to 165 HV at 10 °C/s. This phenomenon can be attributed to the larger structure strengthening effect generated at higher cooling rate. For Cu-bearing steel, a significant peak in Vickers hardness appears at 0.2 °C/s, with the hardness value of about 212 HV, while Cu-free steel only exhibits 156 HV at this cooling rate. In addition, Cu-bearing steel shows a higher level of Vickers hardness than Cu-free steel. It demonstrates that the addition of Cu can effectively strengthen the matrix.

**Figure 4:**
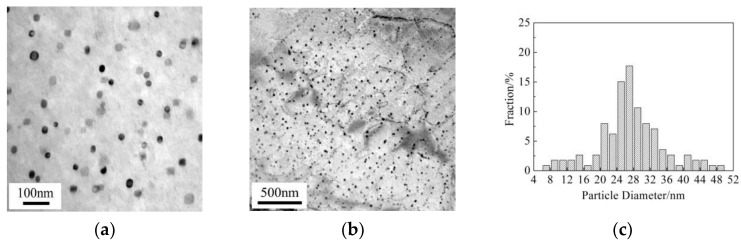

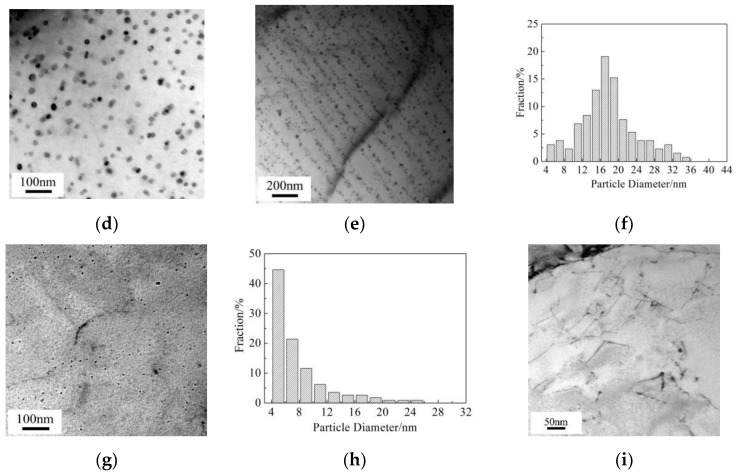
TEM images and histograms showing size distribution of dispersed Cu particles in ferrite for Cu-bearing steel. (**a**–**c**) 0.05 °C/s, (**d**–**f**) 0.2 °C/s, (**g**,**h**) 5 °C/s, (**i**) as-quenched condition in bainite.

To illustrate the micro-mechanism of the above-mentioned Vickers hardness variation for the Cu-bearing steel, Cu precipitates were observed by TEM, as shown in Figure 4. At slow cooling rate of 0.05 °C/s, dispersed Cu precipitates with particle diameter of 26.7 ± 3.6 nm can be observed. Besides, interphase Cu precipitates also exists, in the specimen with the sheet spacing of 78.5 nm and the average particle diameter of 19.8 ± 3.5 nm. At a higher cooling rate of 0.2 °C/s, dispersive precipitation and interphase precipitation occur simultaneously. The particle diameter is 16.5 ± 2.6 nm for dispersed precipitates, the sheet spacing is 60 nm and the average particle diameter is 7.8 ± 4.4 nm for interphase precipitates. As the cooling rate increases to 5 °C/s, Cu precipitates exhibit low number density and tiny size of about 6.8 ± 2.4 nm, simultaneously, no interphase precipitation can be detected. Additionally, Cu precipitates were hardly found in the as-quenched condition, as shown Figure 4i. This suggests that Cu precipitation occurs only when the temperature is below 650 °C in the currently studied Cu-bearing steel.

**Figure 5:**
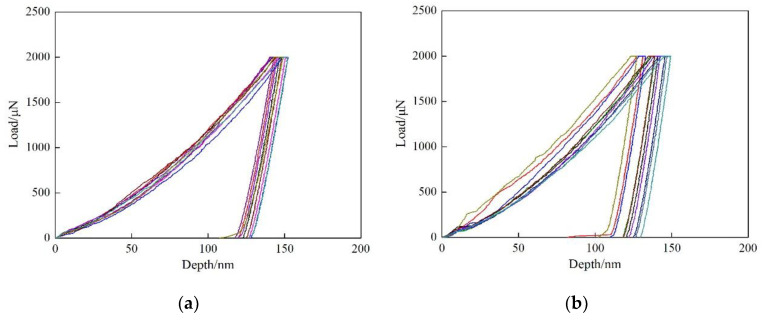

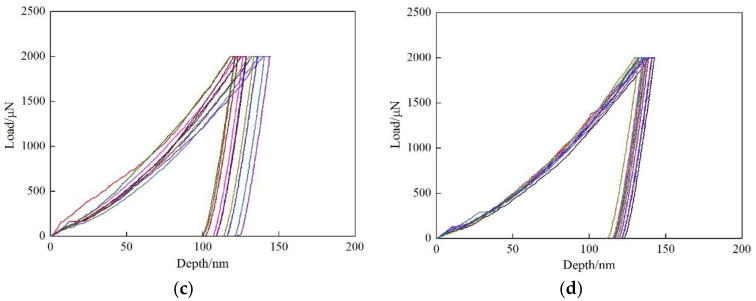
The load-depth curves of the tested steels. (**a**) 0.2 °C/s for Cu-free steel, (**b**–**d**) 0.05, 0.2 and 5 °C/s for Cu-bearing steel, respectively.

In order to further investigate the strengthening effect of Cu precipitates on ferrite phase, nano-indentation experiments were conducted for the two steels, and the results are shown in Figure 5. According to the load-depth curves, the measured indentation depths are within the range of 127~137 nm after unloading for Cu-free steel. While for Cu-bearing steel, the indentation depths are varied between 115~137, 100~29 and 120~131 nm at the cooling rate of 0.05, 0.2 and 5 °C/s, respectively. It is obvious that the indentation depths of Cu-free steel are deeper than those of Cu-bearing steel, which is due to the formation of Cu precipitates during cooling process. Moreover, for Cu-bearing steel, the indentation depths are in wider ranges at the cooling rate of 0.05 and 0.2 °C/s, which can be attributed to the existence of interphase precipitation.

**Figure 6:**
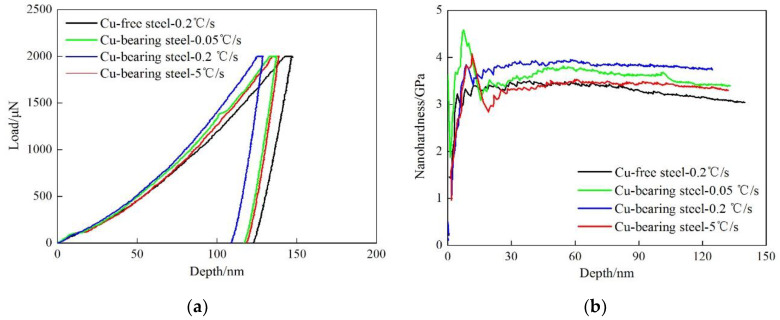
The experimental results of nano-indentation. (**a**) typical load-depth curves, (**b**) nano-hardness-depth curves.

Figure 6 shows typical load-depth curves and nano-hardness-depth curves for the two tested steels. The average indentation depth is 133 ± 3 nm for Cu-free steel. While for Cu-bearing steel, the average value is 124 ± 6, 116 ± 8, and 126 ± 3 nm at the cooling rate of 0.05, 0.2 and 5 °C/s, respectively. In order to directly observe the relationship between nano-hardness and loading depth, the nano-hardness-depth curves can be obtained according to the following equations [27,28].

(1) $$H=\frac{P}{A}$$

(2) $$\begin{array}{cc} A= & 24.5h^{2}+793h+4238h^{1/2}+332h^{1/4}+0.059h^{1/8}\\ & +0.069h^{1/16}+8.68h^{1/32}+35.4h^{1/64}+36.9h^{1/128} \end{array}$$

where *H* is the nano-hardness of the test steel, *P* is the loading force, *A* is the projected area of the contact zone between the indenter and the sample, *h* is the instantaneous depth of the indenter. As shown in Figure 6b, in comparison with Cu-free steel, the nano-hardness-depth curves contain a peak value for Cu-bearing steel. This phenomenon can be mainly attributed to the strong pinning effect of Cu precipitates on the dislocations during the plastic deformation. On the contrast, Cu-free steel contains no Cu precipitates, the dislocations are less hindered. The nano-hardness can get into a stable state immediately after the moving of dislocations. Hence, there is no peak nano-hardness in Cu-free steel.

**Figure 7:**
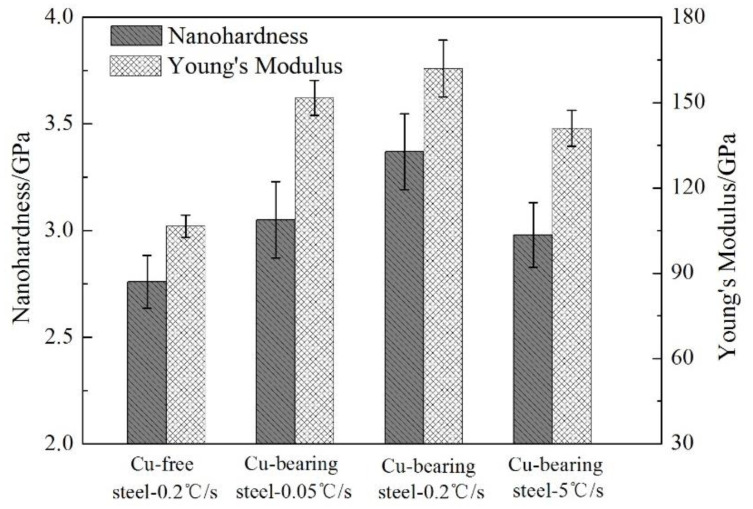
The micro-mechanical properties of Cu-free and Cu-bearing steels.

According to Oliver method and Pharr method [29,30], the nano-hardness and Young’s Modulus of the tested steels can be obtained using Equations (3)–(8), the calculated results are shown in Figure 7.

(3) $$S={\left(\frac{dP}{dh}\right)}_{h=h_{max}}$$

(4) $$H=\frac{P}{A_{c}}$$

(5) $$E_{r}=\frac{S\sqrt{\pi }}{2\sqrt{A_{c}}}$$

(6) $$h_{c}=h-\varepsilon \frac{P_{max}}{S}$$

(7) $$P=\alpha {\left(h-h_{f}\right)}^{m}$$

(8) $$\frac{1}{E_{r}}=\frac{1-\nu^{2}}{E}+\frac{1-\nu_{i}^{2}}{E_{i}}$$

where *S* is the elastic contact stiffness, α and m are the fitting parameters, ε is 0.75, *h_c_* is the contact depth, *h_f_* is the depth after uninstalling, *A_c_* is the projected area of the contact zone between the indenter and the elastic contact region of matrix, *E_r_* is the equivalent elastic modulus, *E* is the elastic modulus, *ν* is the Poisson ratio, *E_i_* is 1114 GPa, *ν_i_* is 0.07.

When the cooling rate is 0.2 °C/s, the nano-hardness and Young’s Modulus of the ferrite matrix are 2.76 GPa and 106.5 GPa for Cu-free steel, while the corresponding values are 3.37 GPa and 162.0 GPa for Cu-bearing steel, respectively. It can be seen that the nano-hardness and Young’s Modulus of Cu-bearing steel are increased by 0.61 GPa and 55.5 GPa compared to those of Cu-free steel, respectively. Comparison of these results indicates that the addition of Cu can effectively strengthen the ferrite matrix. Moreover, for Cu-bearing steel, the nano-hardness and Young’s Modulus increase at first and then drop continuously as a function of cooling rate, sharing the same variation law with Vickers hardness.

## 4. Discussion

### 4.1. Influence of Processing on Cu Precipitation

It has been found that the maximum solubility of Cu in austenite is 2.1 wt% at 850 °C. Therefore, the heating temperature (1100 °C) used in this study is high enough to keep all Cu in solution. It can be concluded from Figure 4i, due to the fast cooling rate of 20 °C/s, Cu precipitation is suppressed at temperature above 650 °C. During the following cooling process, solute Cu will come out of solution to form Cu precipitates when the temperature is below 650 °C. The alloying elements are dynamically diffused between austenite and ferrite during cooling process, leading to different histological types and Cu precipitation behaviors. At a slower cooling rate of 0.05 and 0.2 °C/s, Cu precipitation occurs via two precipitation mechanisms, i.e., dispersive precipitation and interphase precipitation [14,15]. During the nucleation and growth of ferrite grains, interphase precipitation can form at the moving austenite/ferrite phase interface. The sheet spacing and particle size of interphase precipitates are mainly determined by phase transition temperature. As the cooling rate increases, the phase transition temperature decreases, leading to a reduction on sheet spacing and particle size. In addition, at a higher cooling rate of 5 °C/s, because the migration rate of austenite/ferrite interface is too fast, no Cu precipitation is found to occur by interphase precipitation mechanism. Thus, Cu precipitation occurs only by means of dispersive precipitation. Besides, the reduction of undercooling and strengthening of atom diffusion contribute strongly to the fully nucleation and growth of Cu precipitates at slower cooling rate of 0.05 and 0.2 °C/s. As the cooling rate increases to 5 °C/s, the diffusivity of Cu atoms decreases, leading to the suppression of the nucleation and growth of Cu precipitates.

### 4.2. Influence of Processing on Precipitation Strengthening

In order to fully understand Cu precipitation strengthening mechanism, the Cu precipitation strengthening effect can be estimated by Russell–Brown model [31,32].

(9) $$\sigma_{P}=M\frac{Gb}{L}{\left[1-{\left(\frac{E_{p}}{E_{m}}\right)}^{2}\right]}^{\frac{3}{4}};\;{sin}^{-1}\left(\frac{E_{p}}{E_{m}}\right)\geq 50^{\circ }$$

where $\sigma_{P}$ is Cu precipitation strengthening effect, *M* (=3) represents the Taylor factor, *G* (=80 GPa) represents the shear modulus of the matrix, *b* (=0.25 nm) represents the Burgers vector of the dislocations, *E_p_* represents the dislocation line energy in precipitates, *E_m_* represents the dislocation line energy in the matrix, *L* represents the mean particle spacing in the slip plane, which can be estimated by Equation (10) [31,32].

(10) $$L=0.866/{\left(RN\right)}^{1/2}$$

where *R* represents the average particle radius, *N* represents the corresponding number density. Here, the thickness of the thin-film is considered to be 110 nm, thus *N* = 1.2 × 10^−^^6^/nm^3^, 3.1 × 10^−6^/nm^3^ and 2.2 × 10^−6^/nm^3^ can be calculated for dispersive precipitation at cooling rate of 0.05, 0.2 and 5 °C/s, respectively. N = 1.8 × 10^−^^6^/nm^3^ and 7.2 × 10^−6^/nm^3^ can be calculated for interphase precipitation at cooling rate of 0.05 and 0.2 °C/s, respectively.$\frac{E_{p}}{E_{m}}$ is estimated by Equation (11) [31,32].

(11) $$\frac{E_{p}}{E_{m}}=\frac{E_{p}^{\infty }}{E_{m}^{\infty }}\frac{\log\frac{R}{r_{0}}}{\log\frac{r}{r_{0}}}+\frac{\log\frac{r}{R}}{\log\frac{r}{r_{0}}}$$

where $E_{p}^{\infty }$ and $E_{m}^{\infty }$ represent the energy per unit length of a dislocation in the infinite media, *r* (=2.5b) and *r*_0_ (=1000*r*) are defined as the inner and outer cut-off radii. For the ratio $\frac{E_{p}^{\infty }}{E_{m}^{\infty }}$, a value of 0.62 is used according to Ref. [33].

The calculated results are shown in Table 2. It can be seen that the precipitation strengthening effects caused by interphase precipitation and dispersive precipitation in the specimen with cooling rate of 0.2 °C/s are 211.8 and 182.3 MPa, respectively. These values are decreased by 63.9 MPa and 50.0 MPa at cooling rate of 0.05 °C/s. This suggests that the strengthening effect caused by interphase precipitation is obviously higher than dispersive precipitation at the same cooling rate. Therefore, it can be concluded that the wide ranges of indentation depths in Figure 5b,c are caused by the heterogeneous precipitation strengthening effects.

In the present study, the variation of nano-hardness can be attributed to precipitation strengthening, structure strengthening and solid solution strengthening, among which precipitation strengthening plays a dominant role. Cu atoms can diffuse enough distance to form precipitates, and coarsening of Cu precipitates occurs at slow cooling rate of 0.05 °C/s. This phenomenon leads to a dropping of precipitation strengthening effect. The observation of fine and low number density Cu precipitates at 5 °C/s implies that the precipitation strengthening is weakened, causing a low precipitation strengthening effect of 112.4 MPa. From these facts, it is certain that Cu-bearing steel contains a large number of fine Cu precipitates at the cooling rate of 0.2 °C/s, which can provide the maximum amount of precipitation strengthening. Besides, the variations of calculated precipitation strengthening effects are in good accordance with the experimental results of nano-indention, indicating that this model is feasible for evaluating the strengthening effect.

## 5. Conclusions

1. As the cooling rate increases, the optical microstructure transforms from ferritic structure to ferritic + bainitic structure in the two steels. The addition of Cu can refine the ferritic structure.
2. The Vickers hardness values of Cu-free steel slowly rise with the increase of cooling rate, which can be attributed to the increase of structure strengthening. Due to the Cu precipitation strengthening effect, the Vickers hardness values of Cu-bearing steel are obviously higher than those of Cu-free steel. Cu-bearing steel attains the peak Vickers hardness at the cooling rate of 0.2 °C/s, with the hardness value of 212 HV.
3. Dispersive precipitation and interphase precipitation can be observed at a cooling rate below 0.2 °C/s, while interphase precipitation as well as partial dispersive precipitation can be suppressed at faster cooling rate of 5 °C/s. Dispersed Cu precipitates with particle diameter of 26.7 ± 3.6 nm and interphase Cu precipitates of 19.8 ± 3.5 nm can be observed at cooling rate of 0.05 °C/s, while the corresponding values decrease to 16.5 ± 2.6 nm and 7.8 ± 4.4 nm at cooling rate of 0.2 °C/s. The increasing of cooling rate significantly accelerates the formation and coarsening of Cu precipitates.
4. The indentation depths on different cooling conditions are affected by the nature of Cu precipitation, resulting in the differences in nano-hardness and Young’s Modulus. The nano-hardness and Young’s Modulus of Cu-bearing steel are increased by 0.61 GPa and 55.5 GPa than those of Cu-free steel, respectively, suggesting that the addition of Cu can effectively strengthen the matrix. The experimental results of nano-indentation are in line with Vickers hardness tests.
5. The Cu precipitation strengthening effects are estimated using the Russell–Brown model. The precipitation strengthening effects caused by interphase precipitation are obviously higher than those of dispersive precipitation, leading to a wide range of indentation depths. The variation of the calculated values is in line with the variation of nano-hardness, revealing that this model is suitable for calculating the strengthening effects.

## Figures and Tables

**Table 1:** Chemical composition of the tested steels (in mass%).

No.	Fe	C	Si	Mn	P	S	Ni	Cr	Cu	Al	N
Cu-free	Balance	0.0065	0.21	0.68	0.010	0.004	2.53	0.48	-	0.028	0.0050
Cu-bearing	Balance	0.0050	0.20	0.70	0.010	0.004	2.49	0.47	2.01	0.030	0.0050

**Table 2:** The calculated precipitation strengthening effects on mechanical properties of Cu-bearing steel depending on cooling rates.

Cooling Rate, °C/s	Dispersive Precipitation, MPa	Interphase Precipitation, MPa
0.05	132.3	147.9
0.2	182.3	211.8
5	112.4	-
